# From hospitalization records to surveillance: The use of local patient profiles to characterize cholera in Vellore, India

**DOI:** 10.1371/journal.pone.0182642

**Published:** 2017-08-18

**Authors:** Melissa S. Cruz, Tania M. AlarconFalconi, Meghan A. Hartwick, Aishwarya Venkat, Hanna Y. Ehrlich, Shalini Anandan, Honorine D. Ward, Balaji Veeraraghavan, Elena N. Naumova

**Affiliations:** 1 Sackler School of Graduate Biomedical Sciences, Tufts University, Boston, Massachusetts, United States of America; 2 School of Engineering, Tufts University, Medford, Massachusetts, United States of America; 3 School of Marine Science and Ocean Engineering, University of New Hampshire, Durham, New Hampshire, United States of America; 4 Yale University, New Haven, Connecticut, United States of America; 5 Christian Medical College, Vellore, Tamil Nadu, India; 6 Tufts Medical Center, Boston, Massachusetts, United States of America; 7 Friedman School of Nutrition Science & Policy, Tufts University, Boston, Massachusetts, United States of America; Johns Hopkins Bloomberg School of Public Health, UNITED STATES

## Abstract

Despite availability of high quality medical records, health care systems often do not have the resources or tools to utilize these data efficiently. Yet, hospital-based, laboratory-confirmed records may pave the way for building reliable surveillance systems capable of monitoring temporal trends of emerging infections. In this communication, we present a new tool to compress and visualize medical records with a local population profile (LPP) approach, which transforms information into statistically comparable patterns. We provide a step-by-step tutorial on how to build, interpret, and expand the use of LPP using hospitalization records of laboratory-confirmed cholera. We abstracted case information from the databases maintained by the Department of Clinical Microbiology at Christian Medical College in Vellore, India. We used a single-year age distribution to construct LPPs for O1, O139, and non O1/O139 serotypes of *Vibrio cholerae*. Disease counts and hospitalization rates were converted into fitted kernel-based probability densities. We formally compared LPPs with the Kolmogorov-Smirnov test, and created multi-panel visuals to depict temporal trend, age distribution, and hospitalization rates simultaneously. Our first implementation of LPPs revealed information that is typically gathered from surveillance systems such as: i) estimates of the demographic distribution of diseases and identification of a population at risk, ii) changes in the dominant pathogen presence; and iii) trends in disease occurrence. The LPP demonstrated the benefit of increased resolution in pattern detection of disease for different *Vibrio cholerae* serotypes and two demographic categories by showing patterns and anomalies that would be obscured by traditional methods of analysis and visualization. LPP can be used effectively to compile basic patient information such as age, sex, diagnosis, location, and time into compact visuals. Future development of the proposed approach will allow public health researchers and practitioners to broadly utilize and efficiently compress large volumes of medical records without loss of information.

## Introduction

The vast quantity of data generated by biomedical fields and health care has spawned a whole domain for data science. This emerging field seeks to gain insight from data through analysis with statistical, mathematical, and computational tools [1]. The new data sources and streams may offer solutions to pressing needs for disease prevention in data-rich and resource-poor settings, such as hospitals in developing countries [2]. In fact, hospital-based laboratory-confirmed records, especially in light of increasing digitization, are at the forefront of building reliable surveillance systems for emerging infections and diseases of high concern [3–5]. The focus on laboratory-confirmed hospital records is justified due to standardized testing protocols and broad utilization of uniform case definitions by biomedical diagnostic laboratories, ensuring high data quality, fidelity, and consistency [4, 6]. Such properties are very desirable for modern surveillance as they allow for comparison of records across sources and identify aberrations in various contexts, including infectious outbreaks [3, 4, 7, 8].

Medical records typically include patient characteristics such as date of admission, place of residence, sex and age; these features represent information about time, location and demographics, respectively [9]. These data also offer sufficient quality and granularity through multiple aggregation options for time, location, and demographic properties providing choices with varying levels of detail and complexity [2, 8–11]. Large amounts of laboratory records can be analyzed through data aggregation by a specific data feature or compression factor. Any trends or patterns, revealed by this process can be formally tested for statistical significance, reviewed for clinical importance, and potentially inform actions, including prevention or intervention programs [4, 6, 8]. Thus, in our communication, we rationalize and present a new data compression approach, called a local population profile (LPP), which transforms information into statistically comparable patterns.

We define LPP as the single-unit distribution of a variable of interest for a selected population. The LPP aims to take full advantage of large data repositories for compilation of information at very granular level while preserving data precision. In such situations, the LPP operates on the whole range of plausible and available values for the variable of interest or compression factor using the smallest reasonable unit of aggregation. The LPP thus enables a more refined statistical analysis. In this tutorial, we illustrate how to construct and analyze LPPs using readily available medical records with age distribution as the compression variable. We selected age as the compression variable due to the uniformity of collection in patient data and the biological significance in relation to a health condition.

Medical records currently collect data on patients’ age quite reliably. Patient age can indicate the subset of a population that is susceptible to specific infections and indicate changes in disease patterns and herd immunity [12]. For example, the average age of rubella infection changed from 10–19 years to 15–29 years due to the introduction of vaccination in urban areas of Brazil [13]. Another example is the increase in the proportion of people 20 years or older affected during a widespread measles outbreak over time from 17% in 2008 to 23% in 2009 and 38% in 2010 in France [14]. These examples show changes in herd immunity through characterization of disease prevalence by age, time, and place of residence. Thus, coupling attributes such as age, disease, time of event, and place of residence can provide useful information for characterizing or detecting changes in disease patterns.

The primary analytical method used to analyze the relationship between disease and patient age lacks precision. Traditionally, population is stratified into age categories and disease patterns are analyzed for each of those categories. One such example is the division of the population into three age categories: children, adults, and elderly [15–17]. These broad categories allow researchers to identify large-scale changes in disease incidences or mortality across a population or a subset of the population [17]. However, broad age groupings do not allow for the detection of differences within each group, often resulting in loss of data precision and resolution, leading to difficulties in accurately assessing, characterizing, and estimating disease rates and populations at risk.

In this tutorial, we explore the use of single-year age distribution or LPP in place of age categorization to demonstrate how patient characteristics can be used to describe disease incidence for a group of patients sharing a reported place of residence. To examine the relationship between patient’s disease, age, and sex using LPPs, we abstracted laboratory confirmed cholera hospitalization records from the Christian Medical College (CMC) Hospital in Vellore, India. The CMC Hospital is an internationally recognized medical facility with over 8,000 outpatients and 2,000 inpatients a day [18] and has played an important role in documenting the changing landscape of cholera on the Indian subcontinent. Cholera is an acute diarrheal illness caused by toxigenic serotypes of *Vibrio cholerae* (*V*. *cholerae*) [19]. In endemic areas, infection is mostly asymptomatic or results in mild to moderate diarrhea in the majority of cases. However, some individuals, particularly young children can develop severe and rapidly progressive watery diarrhea and dehydration leading to death if treatment is not initiated in time. A recent study reported that approximately 1.3 billion people are at risk for cholera in endemic countries, and an estimated 675,188 cases of cholera resulting in 20,256 deaths occur annually in India [20]. The Department of Microbiology at CMC in Vellore, India provides the major hospital-based laboratory service and has tracked the progression of *V*. *cholerae* O139 since its first detection, documenting the virtual absence of O1 *V*. *cholerae* during 1992–1993, the eventual reappearance of serotype O1 in late 1993, and the concurrent prevalence of O1 and O139 serotypes in Vellore since then [21]. Out of 200 serotypes of *V*. *cholerae*, serotypes O1 and O139 cause severe and wide spread epidemics [19]. Thus, readily available data allows us to demonstrate the usage of LPP to detect the differences in age distributions of pathogenic cholera strains.

Cholera is endemic in India [22] and outbreaks occur regularly with peaks in July and August [23]. Seasonality of cholera is partially controlled by environmental and climatic factors [21] in both endemic and epidemic scenarios [24]. Emerging changes in climate may thus influence temporal fluctuations of cholera and increase frequency and duration of outbreaks [21, 25, 26]. In Vellore, cholera is of particular interest due to the burden its environmental reservoir possess on the local healthcare system, and opportunities for effective community-based preventions [22]. The monitoring of cholera, caused by ingestion of *V*. *cholerae* from contaminated food or water, is expensive and labor intensive [27]. Clearly, a cost-effective approach to outbreak detection on the local level via hospital-based surveillance would provide valuable information for disease monitoring. As highlighted by the World Health Organization and International Health Regulations, relevant information produced from a surveillance system include but are not limited to: i) detection of epidemics or outbreaks, ii) estimation of the geographic and demographic distribution of disease, iii) detection in changes in infectious and environmental agents [28–31]. We see future capabilities of the LPP for illustrating all three main surveillance capacities. However, this work focuses primarily on illustrating the ability of hospital records expressed as LPPs as a tool for detecting differences in disease patterns by sex, causal agent, and trend over time.

In this tutorial, we provide step-by-step instructions on how to compare single-year age distributions or LPPs across demographics, cholera serotypes, and time with the Kolmogorov Smirnov (KS) test, to estimate disease rates using raw and adjusted census data, and to illustrate differences and changes in patient demographics through novel visualization tools, including multi-panel plots. The specific aim of our work is to offer a tool that allows for visual and formal comparisons of datasets of different sizes, yet preserving the refined resolution of key variables.

## Materials

### Data sources

We abstracted all available hospitalization records of laboratory-confirmed cholera cases from 1992 to 2014 (over 1,900 records) from both paper logs (1992 to 2004) and digital entries (2004–2014) from CMC in Vellore, India. Laboratory confirmation was conducted by the Department of Clinical Microbiology [22]. Stool samples of patients in the Emergency Department or admitted with a history of watery and frequent stools were registered for culture with hanging drop, which was then processed for *V. cholerae* [22]. Only positive results of *V*. *cholerae* were included in our analysis. Each record included patient age, sex, *V*. *cholerae* serotype, place of residence, and hospitalization date. To illustrate the use of LPP, we selected only those cases that listed the city of Vellore as the place of residence (n = 583, representing 41.6% of 1,401 cases reported between 2000 and 2014). All 583 records had hospitalization date, while 5 (0.86%) records were missing age, 1 (0.17%) record was missing sex, and 7 (1.20%) records were missing serotype. Age for children <2 years old was recorded in months and converted into decimal values. For patients 20 years old and older, we observed spikes in the single-year age distributions at 5-year intervals, which indicates rounding in the recording of patient age. We applied corrections for the spikes as detailed in the methods section.

We also used census data from 2011 for the district of Vellore obtained from the Office of the Registrar General and Census Commissioner [32]. LPP applied to the census data also demonstrated spikes at 5-year age intervals suggesting rounding by either the individual or the census collector. Census data adjustment is also further detailed in the methods section describing hospitalization rate calculations.

### Ethics statement

Approvals for data use and analysis were obtained from the Christian Medical College Institutional Review Board and by the Tufts Institutional Review Board. All data analysis was conducted on de-identified records. Disease frequency data is available in S1 Dataset and Vellore census data is available online from the Office of the Registrar General and Census Commissioner [32] for analysis replication.

## Methods

### Construction of local population profile

The LPP is a single-unit distribution of a variable of interest for a selected population, in our example it is equivalent to the single-year age distribution for which we convert frequency units to probability estimates to allow comparison of samples of varying sizes. First, we selected one year as our single-interval for single-year age distribution or LPP. We then generated a frequency table of disease counts by age from the cumulative number of cases over the 15-year period. Disease frequency was then converted into probability units by applying a kernel density estimator on the age datum [33]. A kernel is a non-negative function with mean zero that is applied to each age level and creates density estimates or kernels for each age level [33]. The individual kernels are then coupled to create a smooth curve from minimum to maximum age (*i* = 0 to *N* = 90 for cholera cases) by summing the overlapping individual kernels of each age level with the constraint that the sum under the curve is one [33]. Eq 1 illustrates how the integral was modified to create a probability density function of single-year age levels. The integral of *f(Y)dY* can be interpreted as the overall disease incidence, *Y* from age *i* to *N*:

$$P\left[i\leq Y\leq N\right]=\int_{i}^{N}f\left(Y\right)dY.$$ (1)

The kernel density estimator results in disease incidence probability estimates for each age level. We applied the kernel density estimate method for multiple stratifications of our patient data, which forms the visual aspect of the LPP.

We implemented the kernel density estimate through the readily available density function in R, a freely available statistical software used throughout our analysis [34]. The available parameters for the density method are the following: data from which the estimate is computed, smoothing bandwidth, kernel type, weights for each observation, the number of equally spaced points at which the density is to be estimated, and the first and last point where the density should be computed. Our input data was patient ages, no weights were added to the observations, a Gaussian distribution was selected for the kernel type, and the number of equally spaced points for the grid was selected as 512, the default in R. The function creates kernel density estimates for each 512-grid point ranging from 0 to 90, the first and last point where the density should be computed, which are the minimum and maximum ages found in our data set. We also used default R parameters for the smoothing bandwidth, where the kernels are scaled such that the bandwith is the standard deviation of the smoothing kernel.

Depending on the population at risk, a resulting LPP could have different distributions, which are typically unknown *a priori*. The distribution could be uniform, when people at any age have the same probability to be affected by a disease; or it could be bell-shaped, when there is an age group most frequently observed in a study population; or it could have two or more modes signifying most prominent age groups at risk, for example, children and elderly.

### Comparison of LPP with Kolmogorov Smirnov test

While the visual inspection and estimation of local minimum and maximum values for age are helpful for describing differences and similarities, a formal test comparing distributions solidifies the findings. The use of single-year age distribution allows us to treat LPP as a continuous distribution and formally compare LPPs using the KS test [35], which is a nonparametric test that examines the difference between the two empirical distributions, as in Eq 2:

$$E_{N}=d\left(Y_{i}\right)/N,$$ (2)

where *N* is the total number of ordered data points or maximum age, and *Y*_*i*_ is the number of disease cases for the specific age *i*. The KS test works by calculating the maximum possible difference between two empirical distributions (the Kolmogorov distance) [35]. If the distributions are identical then the Kolmogorov distance is zero [35]. The empirical distributions are judged to be different when the Kolmogorov distance between the two distributions is sufficiently large [35]. The Kolmogorov distance between two distributions is estimated in Eq 3:

$$D=max_{1\leq i\leq N}\;\left(F_{1}\left(Y_{i}\right),\;F_{2}\left(Y_{i}\right)\right),$$ (3)

where *N* is the total number of ordered data points, *Y*_*i*_ is the number of disease cases for the specific age *i* for compared distributions *F*_1_ and *F*_2_. Eq 3 calculates the maximum difference between the two curves, or the test statistic *D*, to decide whether or not to reject the null hypothesis. The null hypothesis states that the two curves being compared represent the same distribution or come from the same distribution [35]. The R statistical software provides test statistics *D* and corresponding p-values for KS test with specific methods described for p-value calculations [34].

The KS test was also used to examine potential differences between the LPP of cumulative number of cases over the 15-year period and LPP of cases for each individual calendar year. An LPP can be constructed to reflect the age distribution among the patients admitted during specific time periods, for example during a specific calendar year or during an outbreak. This type of analysis allows us to assess temporal stability or variability of an age distribution and to detect potential aberrations associated with particular events. By comparing a distribution exhibited in one year to an overall distribution, one can see how a given year or pattern differs or is similar to the rest of the data.

### Calculation of hospitalization rates

We calculated single-year age specific rates using the 2011 Vellore census records and assumed that from 2000–2014 the Vellore population stayed constant. Analyzing the census data, we detected characteristic peaks and dips at every 5-year intervals, especially for older adults. These peaks are likely to reflect rounding in collecting information when census takers recorded person’s age by the nearest age group. If reported values were used directly, the peaks would produce erroneous disease rate estimates. Therefore, we corrected them by applying a dual-weighed interpolation scheme to smooth out and redistribute the values at the single year age resolution. The scheme was implemented in three steps.

First, we redistributed the peaks observed at each 5-year mark using linear interpolation. We applied the interpolation so that the value at each 5-year peak was evenly distributed to the ages between the peaks. Redistribution between each 5-year point was accomplished using Eq 4:

$$a_{b}+\left(a_{b+5}-a_{b}\right)*\frac{e}{5},$$ (4)

where *a*_*b*_ represents the total number of people at a given age *b (b = 0*, *10*, *20*, *…90*) and *a*_*b+5*_ the total number of people at the 5-year mark after *a*_*b*_. Variable *e/5* represents the four steps between each 5-year mark, with *e* ranging from 1 to 4 to represent the four numerical values between *a*_*b*_ and *a*_*b+5*._ After generating those numerical values, we replaced each 5-year peak with the average of the values immediately before and after the peak (Eq 5):

$$\frac{\left(a_{d+1}+a_{d-1}\right)}{2},$$ (5)

where *a*_*d-1*_ and *a*_*d+1*_ represent the total number of people immediately before and after the 5-year mark, respectively. Eq 5 was applied to all *d* ages that are multiples of 5. The equation was not applied to age 0, because census data does not exist before that age. In this manner, we approximated the census values at the five-year mark to be in between the two surrounding points.

Finally, we averaged the results from the two previous steps using segmented normalization. This step adjusts the average of both interpolations so that it corresponds to the total census population. We fitted individual segments *c* = 0–13, 14–23, 15–23, 24–33, …84–90 to correspond with the specific census total from the original census. The age interval corresponds to peak intensity, as it is likely that rounding occurred more often for people of older age. Our population total in this equation is represented by *h*, with *h*_*ori*g_ and *h*_*adj*_ representing original and adjusted census values, respectively. To calculate the multiplication factor for the adjusted census values, we used Eq 6 where *c upper* and *c lower* refer to the upper and lower bound of individual segments:

$$\sum_{c\;lower}^{c\;upper}\frac{h_{orig}}{h_{adj}}$$ (6)

We provide a detailed example of our steps taken to smooth the census population and redistribute the spikes across the ages in S2 Table.

We also noticed peaks at 5-year intervals at the age of 50 in our Vellore patient data from initial histograms during exploratory analysis. Thus, we applied the dual-weighted interpolation scheme, as described above using census data, to correct hospitalization counts for ages 50 and over from our Vellore patient data. After applying the interpolation, we estimated age-specific hospitalization rates, *z*, by dividing the age specific adjusted disease counts by the adjusted age specific census data, *q*_*i*_ with the multiplier of M = 100,000 used to place the rate in units of per 100,000 population.

### Creation of multi-panel plots

To illustrate the temporal changes in population profiles we combined LPP plots, time series, and image plots of disease rates to form a multi-panel plot, as introduced by Chui et al [36]. This multi-panel plot aims to represent the interactions between time, age, and disease occurrence. The LPP will share the age axis with that of the image plot of disease rates, while the time series will share the same monthly axis as a heat map of disease rates.

We now describe step-by-step the construction of the multi-panel plot. The core or center of the multi-panel plot is an image plot, where each square represents counts or rates of disease cases for a selected time interval (daily, weekly, monthly or yearly) on the x-axis and a given age interval on the y-axis (single-year age) for an observed study period. The time series is constructed as a needle-plot where each needle represents the total number of cholera cases or rates for a specific time point. We combined the overall LPP, the time series plot, and the image plot based on common or shared axis to form a multi-panel graph. The heat map shares the time axis with the monthly time series plot. On the left of the heat map is a single-year age distribution, or LPP, sharing the axis that reflects age. Since the *y*-axis of LPP shows frequency, panel b describes the overall frequency of disease counts at a particular age for the heat map. Thus, the multi-panel plot incorporates information shown in an LPP and image plot of disease cases. Visually this multi-panel graph highlights important age-related features of the studied population: the most prevalent ages and the time period when the highest or lowest disease occurrences are observed.

## Results

### Summary statistics and age distribution

Five out of the 583 cholera records had no age information, representing less than 5% of effective and actual size for all data subsets based on sex and cholera serotype (Table 1). Stratified by single-year-age and sex, the minimum age in all subsets is 0 years except in the female group infected with *V*. *cholerae* serotype O139, where the minimum age is 1. The maximum age ranges from 65 to 90 for all subgroups. The positive coefficient of skewness indicates that the mean age is higher than the median age and the distribution is skewed to the right. The coefficients of kurtosis are negative, which indicates that the age distribution spiked in younger patients. Two groups deserve special attention: females infected with *V*. *cholerae* O1 serotype and females infected with the O139 serotype were on average the youngest and the oldest subgroups, respectively.

**Table 1:** Descriptive statistics* of cholera patient ages from Vellore.

Category		Effective Size	Actual Size	Max	Mean	Std	Lower Quantile	Median	Upper Quantile	Skewness	Kurtosis
**Total**		583	578	90	22.8	23.1	2.00	15.0	42.0	0.726	-0.739
	**M**	328	324	90	23.4	25.0	1.56	10.0	47.0	0.719	-0.900
	**F**	254	253	75	22.2	20.5	2.00	20.0	35.0	0.648	-0.722
**O1**		414	412	90	21.3	22.9	2.00	10.0	40.0	0.885	-0.454
	**M**	232	230	90	22.5	25.1	2.00	8.00	46.0	0.835	-0.723
	**F**	182	182	70	19.8	19.6	2.00	15.0	30.0	0.811	-0.473
**O139**		42	40	73	32.3	23.9	6.50	32.5	55.3	-0.001	-1.512
	**M**	21	19	73	39.1	23.7	22.0	48.0	57.5	-0.373	-1.358
	**F**	20	20	65	27.4	22.9	4.50	27.5	43.0	0.248	-1.491
**Non O1/O139**		114	113	78	25.1	22.9	1.25	25.0	43.0	0.468	-0.971
	**M**	68	68	78	22.8	23.9	1.00	12.0	43.0	0.634	-0.968
	**F**	46	45	75	28.6	21.0	6.00	29.0	44.0	0.252	-0.833

* Patients are grouped by sex, serotype, and then serotype and sex using age. Effective size: the total number of records (including NAs); actual size: complete data with all patient age information and demographic information relevant to the group.

**Fig 1:**
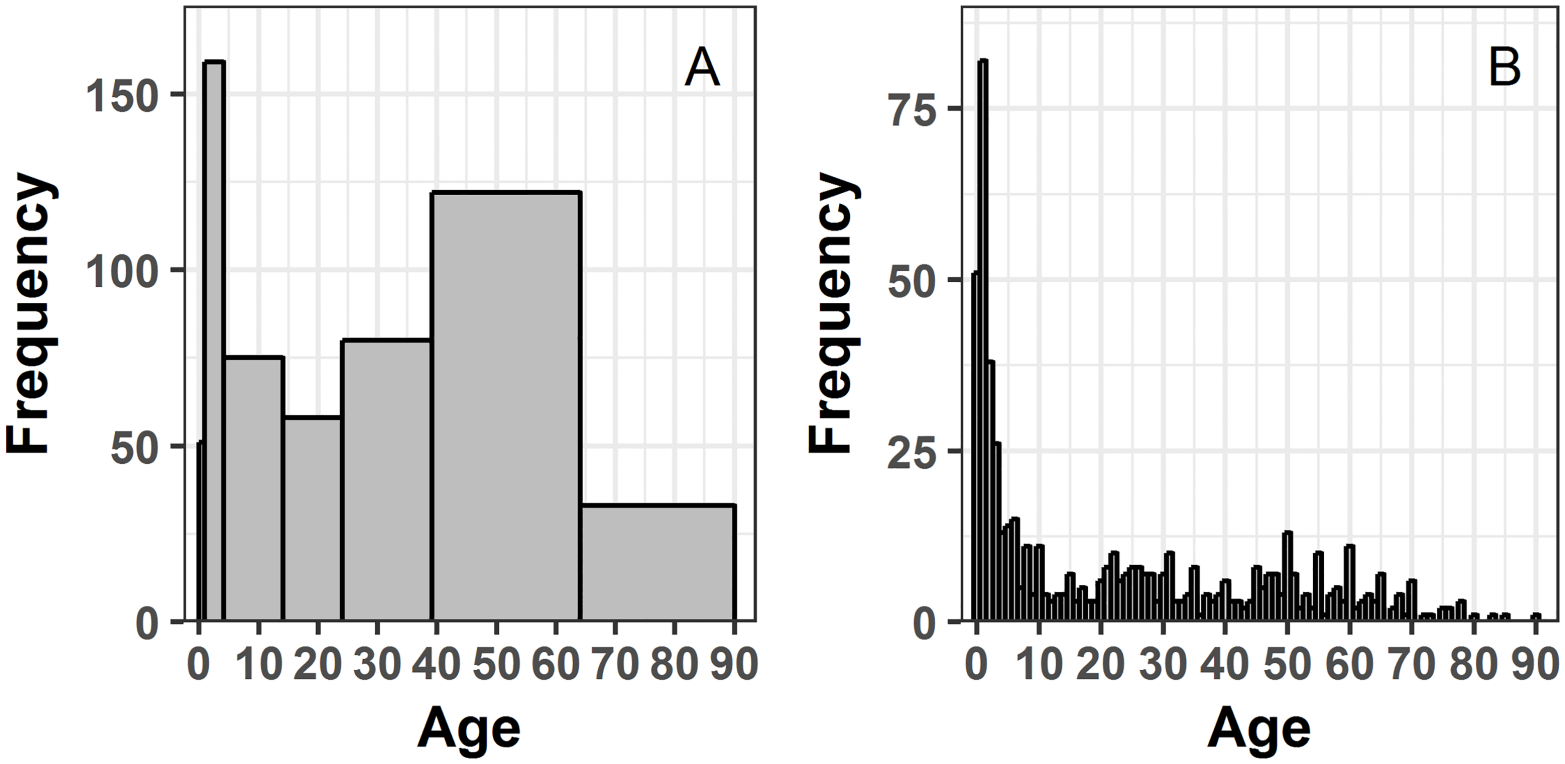
Histograms for age distributions. a) with uneven age intervals; and b) with single-year age interval.

Age distributions are traditionally depicted with histograms or bar graphs with regular (i.e., equally spaced) and irregular intervals. These graphs show the frequency with which values within selected intervals occur in a data set. We used histograms with typically used uneven (<1, 1–4, 5–14, 15–24, 25–39, 40–64, and 65–90) and even (0, 1, 2, 3, etc.) single-year age intervals to demonstrate the effect of age groupings on the same age distribution for the cumulative number of cases over the 15-year period (Fig 1).

A histogram of cholera cases with uneven age intervals (Fig 1a) shows a compact description of age distribution. The highest disease frequency appears within the 1-4-year-old category, decreases concurrently with the 5-14- and 15-24-year-old age groups, picks up in the 25-39-year-old category and again among 40-64-year-old patients and, finally, decreases in the 65 plus category. The histogram of single-year age distribution of cholera cases (Fig 1b) elucidates a different picture: a) the highest disease frequency is among 1-year-old patients with a gradual decline not seen in Fig 1a in the first two age groups; b) disease frequency increases in people aged 20–31 and 45–70, two age ranges that do not match the nearest aggregated groups seen in Fig 1a, 15–24 and 40–65, respectively; c) unusual peaks are seen at 30, 35, 40, 45, 50, 55, 60, 65, and 70 year olds, which is obscured in Fig 1a due to aggregation; and d) case frequency declines steadily after the age of 70, which is not apparent from Fig 1a. We will use LPP plots on the cumulative number of cases over the 15-year period to further explore these features.

### Local population profile construction and comparison

**Fig 2:**
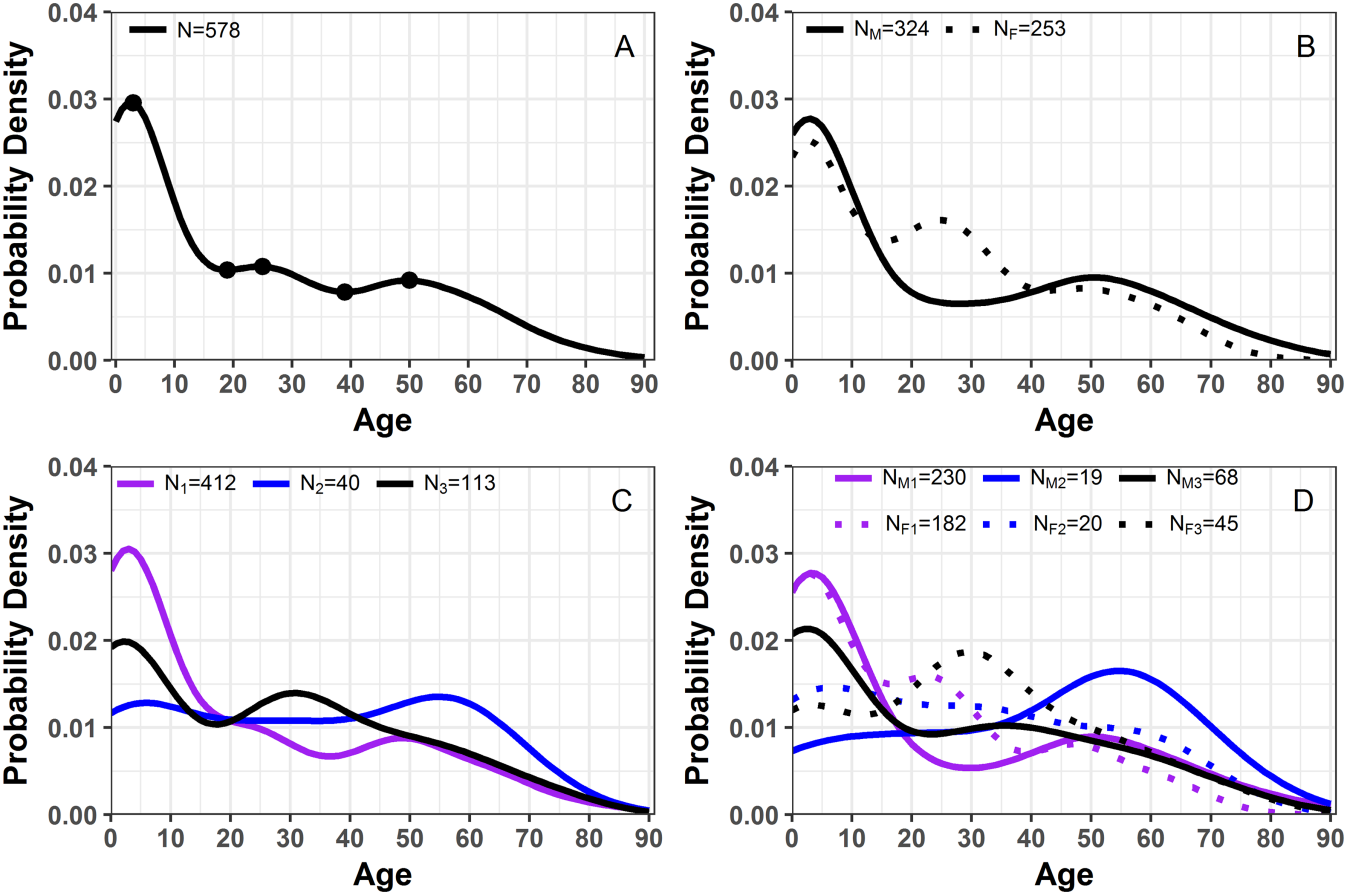
Location-specific patient profile plots for cholera patients. a) plot highlights the inflection points of disease probability of all Vellore patients; b) probability plots by sex with subscripts M and F referring to males and females; c) probability plot by serotype with subscripts 1, 2 and 3 referring to serotype O1, serotype O139, and non O1/O139 serotypes; d) probability plot by sex and serotype with subscripts as a combination from plot b and c. N is the total number of patients.

Visualization of age distribution as LPPs using probability units provides additional details on how disease affects specific populations or ages. Fig 2a shows LPP for the reported Vellore cholera cases for multiple stratifications of the data. By creating LPPs for specific data subsets, we allow for direct comparison of LPP across groups, such as between males and females (Fig 2b), cholera serotypes (Fig 2c), and across sex and cholera serotypes (Fig 2d). In this section, we further demonstrate the use of LPPs in spatial and temporal analysis of disease frequency and rates.

Fig 2a shows LPP plots for 578 Vellore cholera patients as a probability density with respect to age. As expected, the distribution of cholera cases across the age range is not uniform and has characteristic peaks and valleys. Finding those local maximum and minimum values allowed us to characterize the distinct age groups with elevated or reduced disease occurrences. S1 Fig shows that for all subgroups, except O139 males, young children were the most affected.

For the overall distribution (Fig 2a) we see three humps, the largest among children under 4 with a notable decrease after age 2.64. The second hump is among young adults between ages 18.8 to 39.1 years old with a downward slope after age 24.5. The last hump is among adults between 39.1 to 60 years old with a downward trend after age 49.7. The differences from the first and second peak of the humps or local maximums is 21.9 years and 25.2 years between the second and the third. This feature is even more prominent for female patients (Fig 2b), who show a rise in disease frequency starting at age 15.5 until the age of 24.7 and with another peak at the age of 42.6 until the age of 48.3. In contrast, male patients have the highest peak at age of 2.99 with a modest increase between the ages of 28.2 to 50.5. Fig 2c contrasts cholera serotypes, while Fig 2d contrasts all possible permutations of sex and cholera serotype. The male patients infected with serotype O139 (N = 19) exhibited a main increase among older adults while the female patients infected with the same serotype displayed no increase at a particular point but a slight downward slope with a dip around age 70.

We compared multiple stratifications of data for similarity with the KS test in Table 2. We also formally compared annual LPPs with the LPP constructed for all data using the KS test. The results of the comparison are featured in S1 Table, where we see that in year 2000 and 2002 the age distribution differed from that of the rest of the data. Additional information gained from the LPP visualization comes from the confidence interval built using the LPP (S2 Fig). The confidence intervals clearly show where the greatest variability lie on the age spectrum: between children and adults. Teenagers and elderly exhibit the smallest variability. The results suggest that the susceptibility of teenagers and elderly fluctuate the least from year to year (S2 Fig).

**Table 2:** The results of Kolmogorov-Smirnov (KS) test.

Parameter (Sample size)		D-statistic	P-value
**Sex (n)**			
Male (324)	Female (253)	0.119	**0.035**
**Serotype (n)**			
O1 (412)	O139 (40)	0.255	**0.017**
	Non O1/O139 (113)	0.168	**0.014**
O139 (40)	Non O1/O139 (113)	0.205	0.166
**Sex and Serotype (n)**			
O1, Male (230)	O1, Female (182)	0.113	0.149
	O139, Male (19)	0.403	**0.007**
	O139, Female (20)	0.220	0.338
	Non O1/O139, Male (68)	0.107	0.585
	Non O1/O139, Female (45)	0.333	**0.000**
O1, Female (182)	O139, Male (19)	0.447	**0.002**
	O139, Female (20)	0.236	0.269
	O139, Female (20)	0.137	0.312
	Non O1/O139, Female (45)	0.304	**0.003**
O139, Female (20)	O139, Male (19)	0.329	0.242
	Non O1/O139, Male (68)	0.176	0.722
	Non O1/O139, Female (45)	0.139	0.952
Non O1/O139, Female (45)	Non O1/O139, Male (68)	0.248	0.071

Comparison for multiple stratifications of Fig 2 are shown in Table 2. A p-value less than .05 indicates that the compared distributions are significantly different and are shown in bold.

### Hospitalization rates estimation and visualization

**Fig 3:**
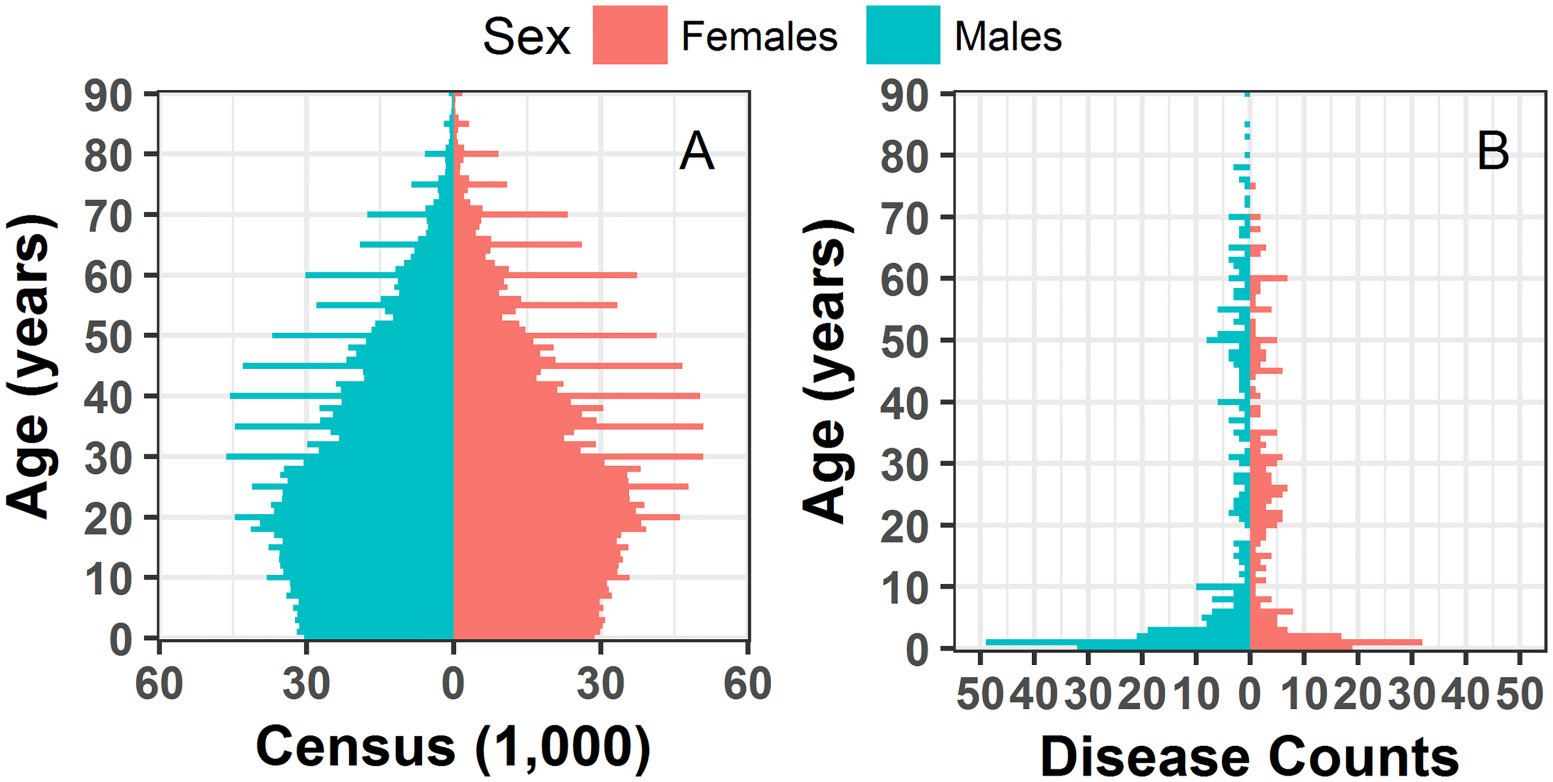
Population and outcome pyramids. a) original census data for Vellore population in 2011 and b) cholera patients, respectively.

**Fig 4:**
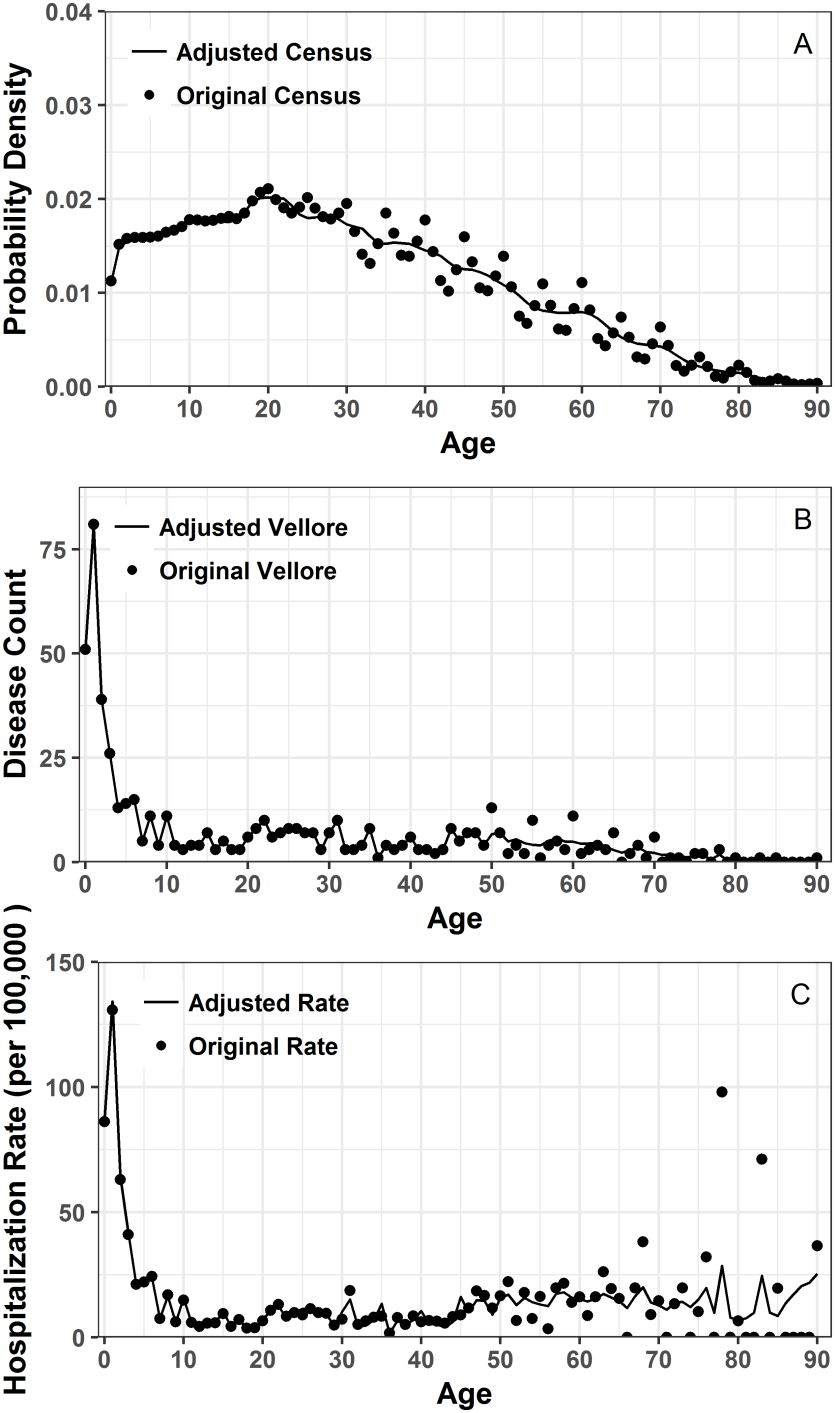
Before and after data smoothing. a) probability density plots of original and adjusted census data for 2011; b) comparison of original Vellore cholera patient disease count by age and adjusted disease count by age; c) original hospitalization rate calculated with original census data and patient values compared with adjusted rate based on adjusted census and adjusted patient data.

The original census data from Vellore 2011 exhibited spikes at 5-year intervals from the age of 20 onward (Fig 3a). We also noticed peaks at 5-year intervals from the age of 50 onwards for Vellore patients (Figs 1b and 3b). The results of the hospitalization rate adjustments are shown in Fig 4a with a density smoother applied. Results of the dual-weighted correction as described for records of patients over 50 can be seen in Fig 4b. Fig 4c shows the difference between hospitalization rates calculated with and without adjustments. Overall, unadjusted rates have higher variability, most likely due to rounding age during data collection.

**Fig 5:**
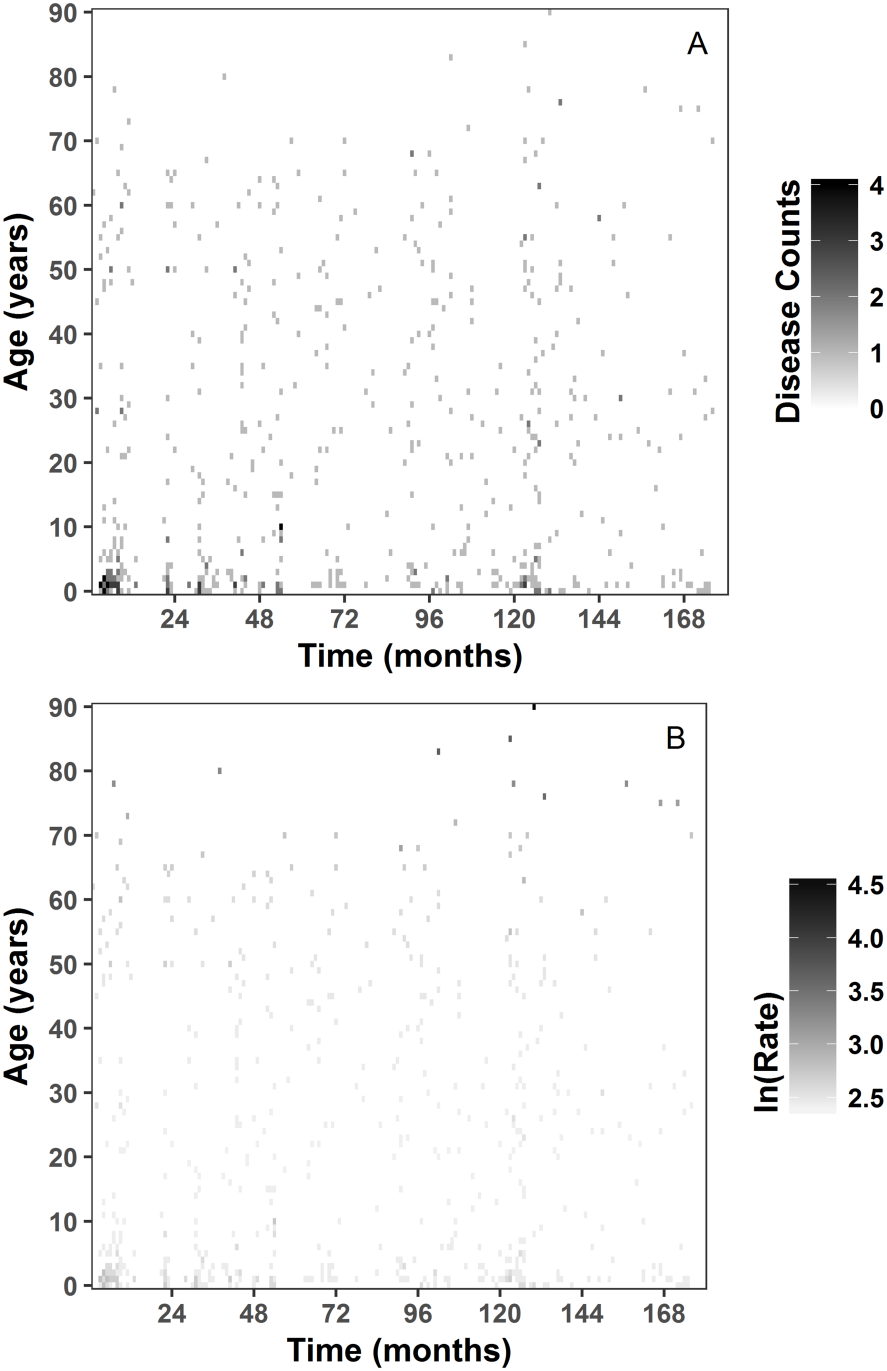
Image plot by single year age intervals on a monthly scale all cases. a) disease counts; b) disease rates are shown as the natural log of rate per 100,000 plus the constant 10 for positive values.

**Fig 6:**
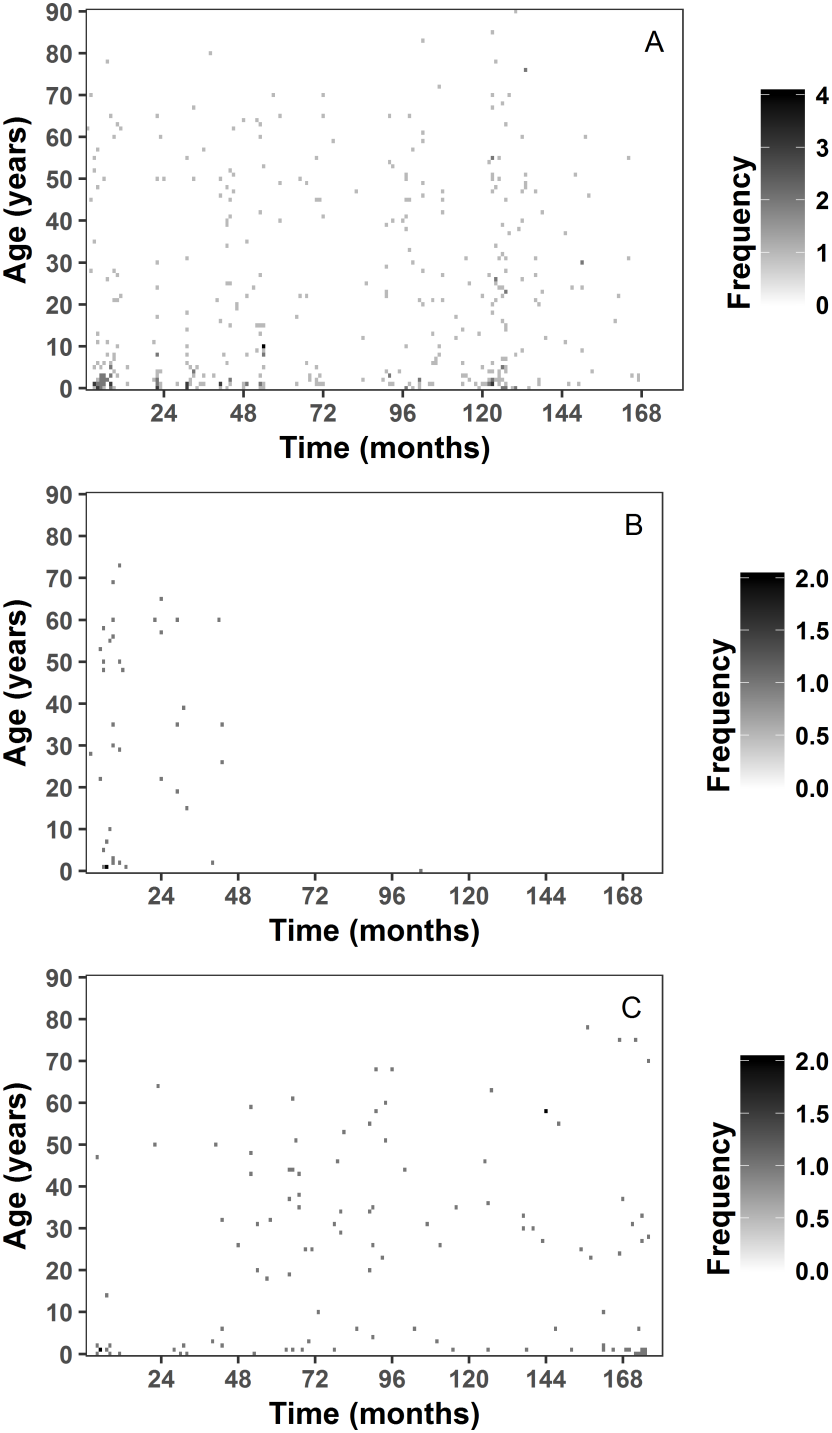
Image plot of serotype specific disease cases by single year age intervals on a monthly scale. a) serotype O1; b) serotype O139; c) Non O1/O139 serotypes.

Fig 5 shows heat maps or image plots of disease incidence and rates over 15 years with months as our time unit. Fig 5a shows disease counts and Fig 5b disease rates for all cholera cases. Fig 6 illustrates the heat map of age specific disease incidence for the specific serotypes of *V*. *cholerae*. The pixels with a deeper color indicate higher disease counts. The heat map of serotype O1 indicate diminishing number of cases of patients after age 70, with cases disappearing after month 168 (Fig 6a). The heat map for serotype 0139 shows that disease cases are present from age 0 to 73, with only one case present after month 48. Finally, cases for non O1/O139 serotypes are present among patients under 10 at first and then after month 36 frequency increased and included a broader age interval. All heat maps show a cluster of pixels with deep color for age 0 to 5 in the first few months of the time series indicating all serotypes were detected in this age group.

**Fig 7:**
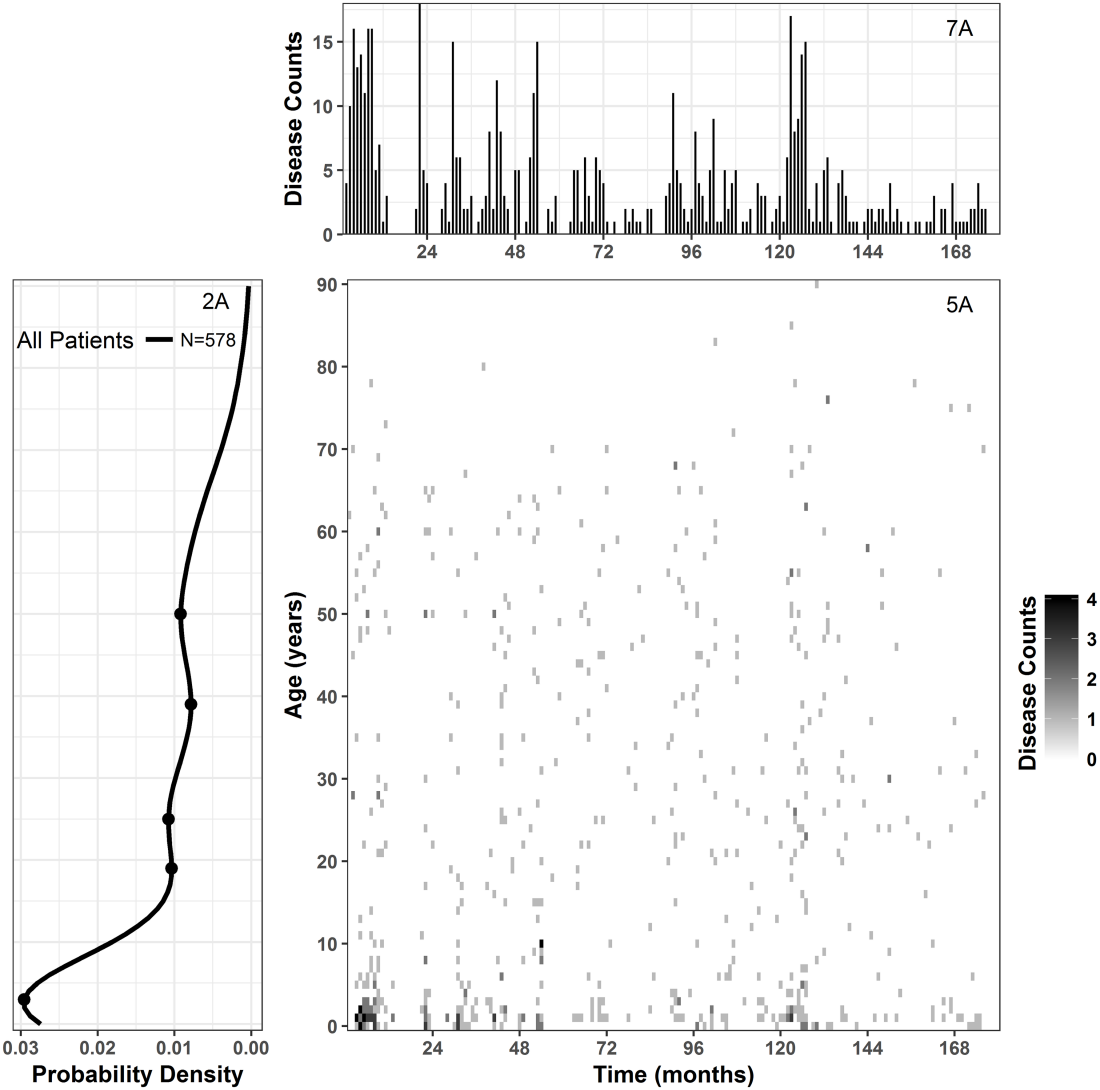
Multi-panel graph of disease. a) time series of disease counts; 5a) image plot of disease cases by time in month and single year age intervals for all patients irrespective of sex and serotypes; 2a) LPP of Vellore.

Each of these heat maps can be supplemented with a time series plot forming a multi-panel plot. The multi-panel plot in Fig 7, consists of an image plot of disease counts, time series, and LPP. The time series at the top of Fig 7 (Fig 7a) is constructed as a needle-plot where each needle represents the total number of cholera cases or rates for a specific time point. The time series shows a downward trend of cholera cases over the past 15 years. Major gaps, indicating lack of recording of disease cases, can be seen roughly between 13 and 23 months, while smaller gaps can be seen throughout the data. Panel 2a on the left of the heat map is a single-year age distribution, or LPP, sharing the age axis with the heat map. The other axis of LPP is showing the frequency, thus panel 2a describes the overall frequency of disease counts at particular age for the heat map. Thus, the multi-panel plot incorporates information shown in Figs 2 and 5 along with a time series.

## Discussion

A local population profile is a single-unit distribution or collection of single-unit distributions representing stratifications of the data. LPPs provide patterns that are statistically comparable for varying sample sizes, while LPP plots allow for visual detection of disease patterns irrespective of sample sizes. In combination, LPP and LPP plots provide an additional tool for exploratory data analysis and visualization, as exemplified in this paper with patient hospitalizations records. Our main results for this specific case study suggest that cholera hospitalization patterns depend on sex and cholera serotype. These patterns by sex and serotype would have been obscured with traditional age bins, illustrated in Fig 1. In comparison to traditional methods such as age categorizations, LPPs allow us to view samples of different sizes with higher granularity due to the use of single-units. Traditional histograms often aggregate available age range to a select few categories, reducing the number of data points available for comparison. Reduction of the effective sample size from 91 data points to 5–7 groups, for example, reduces statistical power needed for various statistical tests; and uneven grouping may distort accuracy. In fact, in our example (Fig 1) the median age calculated using actual data and the mid-points of grouped data would differ by 4.5 years, placing incorrectly the estimated average on young adults (19.5) intstead of teens (15.0). The use of single-year age distribution allows us also to treat LPP as a continuous distribution and apply a broad range of existing statistical tools. Throughout the paper we emphasized the differences between traditional age categorization and the single-unit distribution with respect to visual interpretation.

Our analysis illustrates the advantages of using a single-year age distribution, so called LPP, that can weave basic patient information such as age, sex, disease, pathogenic serotype, location, and time into temporal and demographic visuals. Summary statistics related to study population age are commonly reported in epidemiological investigation. However, without pairing summary statistics with properly binned distributions, the perception of information on age-specific disease incidence can be distorted. For example, Table 1 states patient mean age and standard deviation as 23 ± 23. In a normal distribution, one standard deviation from the mean encompasses 68% of the data. Thus, under the assumption of a normal distribution, 68% of the patients will have an age between 0 and 46 with a center at 23. This interpretation is misleading if the underlying distribution is unknown, as is usually the case. Fig 1b, for example, shows that disease frequency is centered among specific age groups or ages. Additionally, stratification by age intervals may also misrepresent data. The broad age categories can limit the precision and accuracy of the information. For example, in Fig 1 the highest disease frequency is within children between 1–4 years old followed by adults aged 40–64. In comparison, the single-year age distribution in Fig 1b indicates that disease frequency is highest specifically among 1-year-old children followed by infants. Juxtaposing traditional age categories and single-year categories, we see that the specificity in disease frequency is higher using single-unit intervals. Greater specificity is useful for targeted intervention programs and detail description of disease burden within the community. Thus, the single-year age distribution or LPP provides a more detailed picture of disease patterns.

We explored the patterns in disease incidence by converting our LPP from counts to probability units using a density smoother, allowing comparison for samples of varying sizes. To minimize the loss of information, we applied the smoother on a refined grid, imitating a single-year age distribution, so it removes noise and highlights peculiar behaviors. In the case of age of Vellore cholera patients, the noise is the 5-year peaks seen in Fig 1b and in the outcome pyramid in Fig 3b. We suspect that common practice for rounding reported age to the nearest semi-decadal break is the reason for the regular peaks. Smoothing of these peaks allowed for visual detection of three humps (among other patterns) as shown in Fig 2a. With the single-year age distribution, we can clearly observe the 5-year intervals of peaks in the data, whereas 5-year or 10-year bin stratification would have masked the spikes. The higher level of granularity achieved with single-year age distribution or LPP prompts practical recommendations for improving primary data collection.

In addition to smoothing noise, conversion of LPPS as probability units allows for normalization of data of varying sizes. Normalization of distribution with a density smoother allow us to formally compare subsets using KS test as seen in Table 2. This approach guides the generation of new research questions. For instance, do the different strains infect children at the same rate? In Fig 2c we saw that *V*. *cholerae* serotype O1 was the most prevalent serotype in general, yet O1 and non O1/ O139 serotypes have the largest burden in young children. We can test such hypothesis by comparing the age distribution of each serotype with KS test. In Table 2, we see that age distributions of O1 and O139, and O1 and non O1/ O139 are likely to have different patterns, where strain O1 affects patients differently than O1 and O139 with respect to the patient age. This supports the hypothesis that each serotype has a unique age pattern, which might be indicative of age-specific susceptibility for particular serotypes.

The detection of serotypes changes over time. We illustrated the temporal changes with the image plots (Fig 6): for serotype O1, frequency of cases diminished for all ages over time. In comparison, serotype O139 was most dominant in the first 3 years of the study period and almost disappeared after the fourth year of our time series, except for one isolated case. Non-O1/O139 serotypes also show a specific age and temporal pattern: in the first year of our time series, non-O1/O139 serotypes were observed in children under 10 years old and dropped off until it picked back up later. LPPs combined with image plots expand substantially the functional capacity of data analysis.

The LPP plots can also trigger new research questions. For example, coupling knowledge of cholera’s infectious nature and of traditional intergenerational households in India, we suspect that the three humps seen in Fig 2A at approximately age 1, 25 and 50 years (presumably for a child, adult and grandparent) may reflect person-to-person spread of infection within a household. An infected person in a home is likely to spread the disease to caretakers or relatives [37], thus leading to infection in three distinct generations. Given the difference between LPP for males and female seen in Fig 2B, we can also hypothesize that adult women are more likely to be infected than adult men due to their traditional role as caregivers in their households [38, 39].

Difficulties with the LPP approach are encountered in the implementation of the density function in R, and in data quality and interpretation. Due to left censoring at age 0 it was difficult to achieve the sum of the area under the density curve as one. Thus, interpretation of the probability units is limited because the sum of probability units does not equal one. Another challenge is the quality of original data available, as illustrated in the rounding of ages in our original dataset. Interpretation of results from the density smoother is dependent on the accuracy in primary data collection. Higher accuracy in data is likely to increase researcher’s ability to gain useful knowledge from an LPP. Even though the density method is imperfect and variations of the application need to be further explored, valuable information can be gleaned from visualization of an LPP.

The advantages of the single-unit distribution come from the application and utilization of the LPP in characterizing the relationship between disease and patient demographics. Early work by Cohen et al [40], shows the benefit of single-year distributions in quantifying the burden of diseases in the elderly, and the disadvantage of using traditional approaches, like binning. His work illustrates trends and anomalies were obscured when binning age distributions in age categories such as five, ten, or twenty-year age intervals for people aged 65–100. Other examples include the use of single year rates to study the excess of malaria episodes and trends in premature mortality, although for a narrow age range such as 0–20 [41] and 25–64 [42], respectively. We argue that replacing actual age with arbitrary selected categories is likely to distort accuracy and while data aggregation might be seen as a tool to protect confidentiality, little benefits is achived by age binning [43]. In this paper, we applied LPPs as a compression factor covering the whole biologically plausible age range. The proposed approach can be expanded to determine aberrations and complex distributional forms for a broad variety of biomedical, demographic, environmental and socio-economic variables collected in large-scale projects. With increasing volumes of available medical records, LPPs may offer accurate, precise, and highly detailed information in a compact and comprehensive manner.

## Conclusion

Our first implementation of the LPP to hospitalization records has demonstrated the benefit of increased resolution in pattern detection of disease over time for different cholera serotypes and two demographic categories. These patterns revealed information that is typically gathered from surveillance systems such as, i) detection of trends and other temporal changes, ii) estimation of the demographic distribution of diseases and identification of a population at risk, iii) detection of changes in the dominant pathogen presence. Detection of outbreaks can be further explored from the time series plot featured in the multi-panel plot, while the demographic distributions highlight the changes in hospitalization rates. Overall, LPP and LPP plots provide a new tool to visualize age distribution of disease with higher accuracy and precision than traditional methods, and add value in the characterization of the interaction among disease, age, and time. Future development of this approach will allow researchers and public health practitioners to broadly utilize and efficiently compress large volumes of medical records without loss of information.

## Supporting information

S1 Dataset Age distribution and time series data of hospitalization records. (XLSX)

S1 Fig Line graph of inflection points from Fig 2. LPP inflection points from Fig 2 are represented by the star symbol with circles distinguishing local maximums. Each line represents a different group. The order is the following: overall group inflection points, and inflection points for males (M) and females (F) within that group. Thus, we show inflection points for males and females for the overall population and by serotypes in our data set.
(PDF)

S2 Fig LPP of Vellore with 95% confidence interval. Visual inspection shows that the smallest confidence intervals is for teenagers and elderly groups with the highest confidence interval among children.
(PDF)

S1 Table The results of Kolmogorov Smirnov test for multiple years. (PDF)

S2 Table The three steps in readjusting census data. (PDF)
