# Supplementary figures and images for: From hospitalization records to surveillance: The use of local patient profiles to characterize cholera in Vellore, India
Source: PLoS One. 2017 Aug 18;12(8):e0182642. doi: 10.1371/journal.pone.0182642 (PMC5562306; doi:10.1371/journal.pone.0182642)

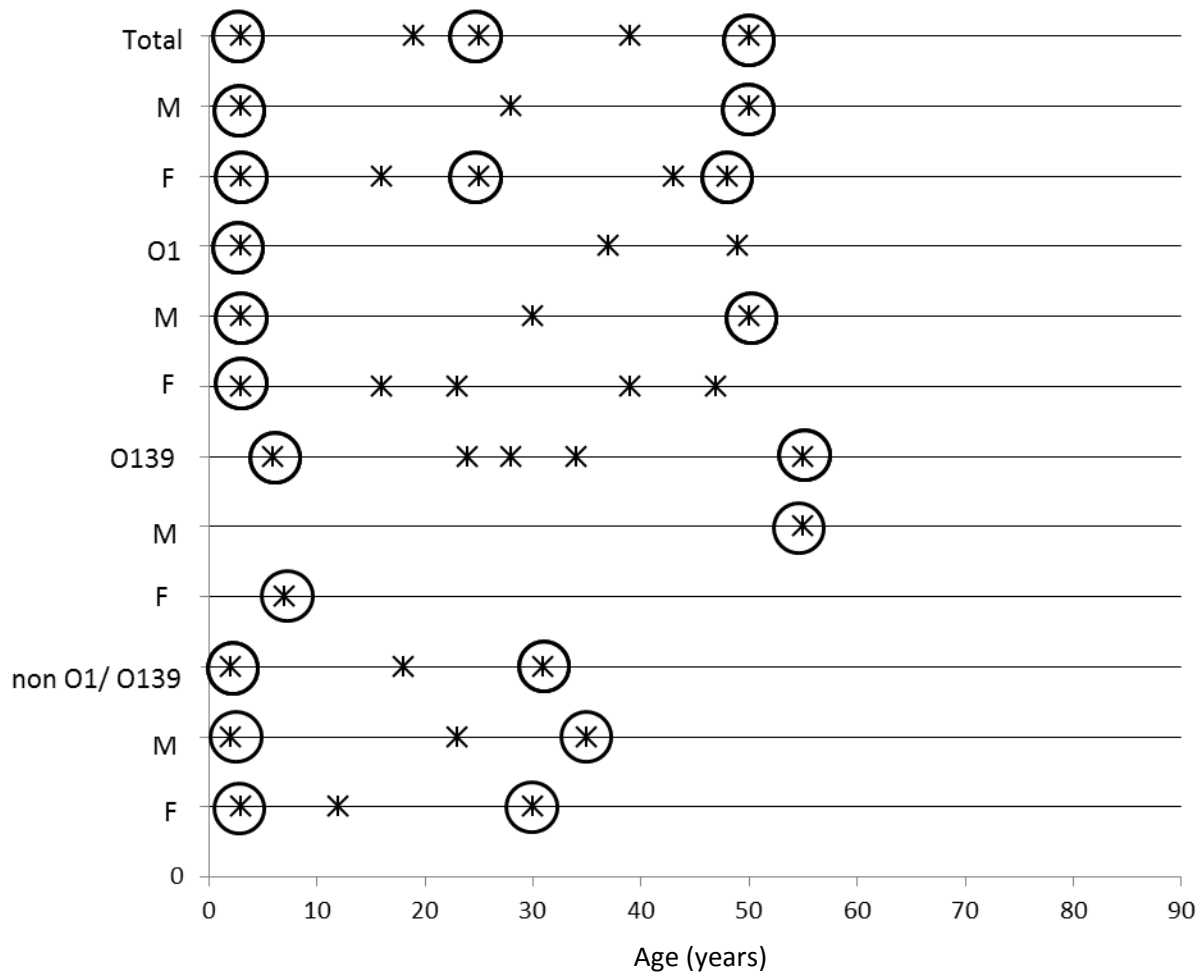

Supplement: S1 Fig — LPP inflection points from Fig 2 are represented by the star symbol with circles distinguishing local maximums. Each line represents a different group. The order is the following: overall group inflection points, and inflection points for males (M) and females (F) within that group. Thus, we show inflection points for males and females for the overall population and by serotypes in our data set. (PDF) [file pone.0182642.s002.pdf]

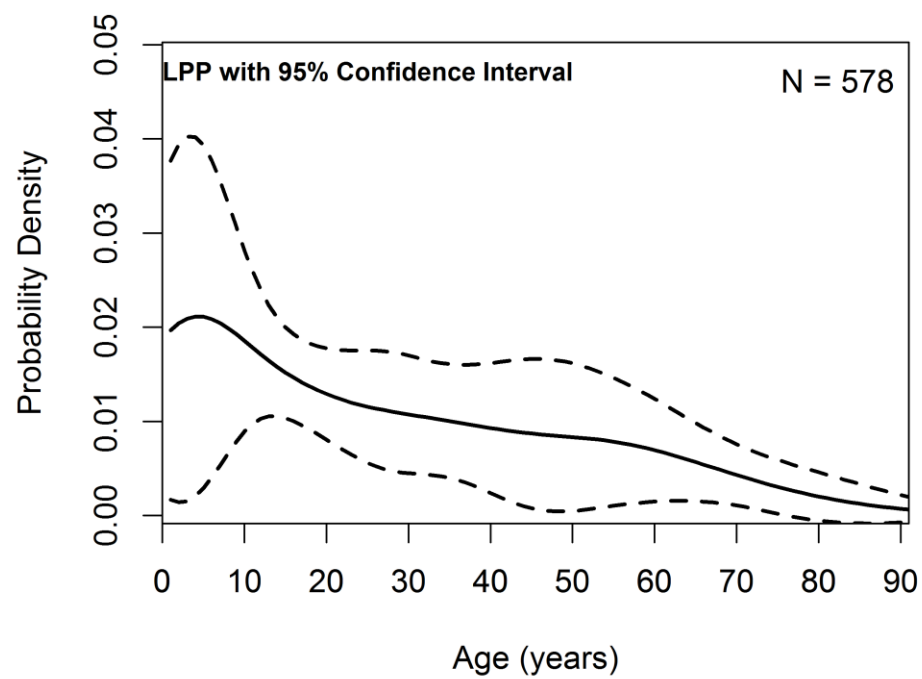

Supplement: S2 Fig — Visual inspection shows that the smallest confidence intervals is for teenagers and elderly groups with the highest confidence interval among children. (PDF) [file pone.0182642.s003.pdf]
