# Supplementary material for: From hospitalization records to surveillance: The use of local patient profiles to characterize cholera in Vellore, India
Source: PLoS One. 2017 Aug 18;12(8):e0182642. doi: 10.1371/journal.pone.0182642 (PMC5562306; doi:10.1371/journal.pone.0182642)
Supplement: S1 Table — (PDF) [file pone.0182642.s004.pdf]

S1 Table. The results of Kolmogorov Smirnov test for multiple years.

Year Series	Number of Patients within Year Series	Vellore total without year series	D Statistic	P value
1	111	467	0.194	**0.002**
2	32	546	0.128	0.701
3	41	537	0.270	**0.008**
4	46	532	0.134	0.431
5	44	534	0.186	0.119
6	37	541	0.167	0.286
7	11	567	0.287	0.336
8	34	544	0.189	0.201
9	46	532	0.161	0.221
10	20	558	0.299	0.063
11	83	495	0.076	0.809
12	25	553	0.261	0.077
13	17	561	0.175	0.695
14	16	562	0.110	0.992
15	15	563	0.315	0.110
