# Supplementary material for: From hospitalization records to surveillance: The use of local patient profiles to characterize cholera in Vellore, India
Source: PLoS One. 2017 Aug 18;12(8):e0182642. doi: 10.1371/journal.pone.0182642 (PMC5562306; doi:10.1371/journal.pone.0182642)
Supplement: S2 Table — (PDF) [file pone.0182642.s005.pdf]

S2 Table. The three steps in readjusting census data.

Age	Original Census Data	Step 1	Step 2	Step 3
0	59208	59208	59208	58634.9
1	61911	60008	61911	60369.4
2	61920	60808	61920	60770.0
3	63371	61608	63371	61884.6
4	61462	62408	61462	61335.5
5	63208	63208	61466	61733.6
6	61470	65366.6	61470	62804.4
7	66293	67525.2	66293	66261.4
8	64970	69683.8	64970	66675.2
9	64672	71842.4	64672	67596.5
10	74001	74001	66397.5	69519.7
11	68123	73892.2	68123	70320.2
12	69086	73783.4	69086	70743.2
13	70189	73674.6	70189	71235.5
14	69517	73565.8	69517	68572.6
15	73457	73457	68781	68167.7
16	68045	76922.6	68045	69475.9
17	70805	80388.2	70805	72459.5
18	80711	83853.8	80711	78867.8
19	77777	87319.4	77777	79122.6
20	90785	90785	75768.5	79820.9
21	73760	90452.2	73760	78698.9
22	76106	90119.4	76106	79663.7
23	70892	89786.6	70892	77005.4
24	70723	89453.8	70723	70412.9
25	89121	89121	70160.5	70019.3
26	69598	90756.6	69598	70491.1
27	70843	92392.2	70843	71757.4
28	72763	94027.8	72763	73320.4
29	61326	95663.4	61326	69011.8
30	97299	97299	57413	68010.6
31	53500	96927	53500	66127.0
32	58882	96555	58882	68329.3
33	45871	96183	45871	62446.2
34	49612	95811	49612	58601.5
35	95439	95439	53030.5	59829.1
36	56449	95528.2	56449	61242.6

37	50737	95617.4	50737	58976.8
38	57910	95706.6	57910	61903.3
39	46815	95795.8	46815	57468.2
40	95885	95885	45496.5	56972.9
41	44178	94639.4	44178	55939.6
42	46405	93393.8	46405	56335.1
43	35128	92148.2	35128	51288.8
44	36223	90902.6	36223	48728.8
45	89657	89657	39460	49492.1
46	42697	87414.2	42697	49873.2
47	37580	85171.4	37580	47052.1
48	41978	82928.6	41978	47878.2
49	34192	80685.8	34192	44034.1
50	78443	78443	32782.5	42634.1
51	31373	75029.2	31373	40785.3
52	29479	71615.4	29479	38750.7
53	22295	68201.6	22295	34688.4
54	26594	64787.8	26594	32379.8
55	61374	61374	27592.5	31524.0
56	28591	62627.2	28591	32321.8
57	20301	63880.4	20301	29828.4
58	23121	65133.6	23121	31271.7
59	21579	66386.8	21579	31169.4
60	67640	67640	22316	31874.6
61	23053	63149.8	23053	30544.7
62	18499	63149.8	18499	28931.1
63	15300	58659.6	15300	26206.5
64	15425	54169.4	15425	22951.2
65	45189	45189	15146	19897.6
66	14867	44337.6	14867	19524.8
67	10151	43486.2	10151	17688.8
68	10480	42634.8	10480	17516.5
69	11029	41783.4	11029	17416.8
70	40932	40932	11290	17222.1
71	11551	36641.2	11551	15893.1
72	7470	32350.4	7470	13132.2
73	5077	28059.6	5077	10928.0
74	6159	23768.8	6159	9574.1
75	19478	19478	6194.5	8212.8
76	6230	18597.2	6230	7942.3
77	3093	17716.4	3093	6657.0

78	3061	16835.6	3061	6365.0
79	3708	15954.8	3708	6290.2
80	15074	15074	3773	6029.2
81	3838	13080.6	3838	5412.3
82	1974	11087.2	1974	4178.3
83	1405	9093.8	1405	3358.6
84	1750	7100.4	1750	2875.0
85	5107	5107	5107	3317.9
86	1809	4631.8	1809	2092.3
87	948	4156.6	948	1658.2
88	611	3681.4	611	1394.4
89	817	3206.2	817	1306.9
90	2731	2731	742.5	1128.3
Total				
	3930156	5709732.6	3488253.5	3930156
